# Pharmacokinetic data in pregnancy: A review of available literature data and important considerations in collecting clinical data

**DOI:** 10.3389/fmed.2022.940644

**Published:** 2022-10-04

**Authors:** Paola Coppola, Essam Kerwash, Janet Nooney, Amro Omran, Susan Cole

**Affiliations:** Medicines and Healthcare Regulatory Agency, London, United Kingdom

**Keywords:** pregnancy, pharmacokinetics, clinical trials, regulatory, ADME

## Abstract

Pregnancy-related physiological changes can alter the absorption, distribution, metabolism and excretion of medicines which may affect the safety and efficacy of the medicines administered in pregnancy. Pharmacokinetic data can thus be instrumental in supporting dose adjustments required in this population. This review considers the availability of published pharmacokinetic data for over 200 medicines of interest for use in pregnancy in the UK, to identify whether sufficient data currently exists, in principle, for any medicine or group of medicines to support dose adjustments to maintain maternal health through pregnancy. Very limited data was found for many of the medicines of interest. Nevertheless, well documented, large changes of exposure for some drugs, where data is available, highlights the urgent need to collect more data of good quality to inform appropriate doses, when needed, in this population. In addition, clinical study methodology can have an impact on the usefulness of the data and key clinical design aspects are highlighted for consideration in future clinical study design.

## Introduction

The benefit-risk decision to inform medicine use in pregnancy relies on the available information about the effect of the medicine on pregnancy outcomes and the effect of pregnancy on the medicine, i.e., the medicine's ability to maintain maternal health during pregnancy. There is, however, limited scientific information to support the safe and optimal use of medicines in pregnancy, and the data available to inform pregnant women about drug safety and efficacy are considered inadequate (1). The lack of information appears to encompass every aspect of pharmaceutics, including limited pharmacokinetic (PK) and pharmacodynamic (PD) information during and after pregnancy to ensure proper dosing.

Several physiological changes occurring during pregnancy may affect the way the body normally deals with administered medicines, leading to possible changes in the PK and PD of medicines as pregnancy advances. Possible changes in the absorption, distribution, metabolism and excretion processes of medicines may result either in increased or decreased blood levels of medicines during pregnancy compared to non-pregnant individuals (2), with consequences for both the mother and baby. Particularly, decreased drug blood levels may lead to loss of efficacy and reduced control of maternal health which may affect fetal health and/or development, whereas increased levels could result in exposing the mother and the fetus to unnecessarily high doses, increasing the risk of adverse effects. To avoid this, some dosing regimens may need adjustments to achieve efficacious levels to maintain maternal health in pregnancy. However, optimal dosages are not usually investigated in pregnant women, PK changes are unknown or poorly characterized for most medications, and it is unknown whether a single dosing regimen will be sufficient throughout pregnancy or whether one or several dose changes would be required as pregnancy advances (3).

The European Medicines Agency (EMA) and US Food and Drug Administration (FDA) recommend that, when possible, PK and PD studies should be conducted in pregnant women to understand better how pregnancy affects the blood levels of medicines commonly used, particularly during the first trimester, and to develop evidence-based guidance for dose and frequency of administration for use in pregnancy (4, 5). Nevertheless, at the time of licensing, this data is not available and most new medicines in the European Union advise avoiding use in pregnancy because of a lack of sufficient data in pregnant women.

Where there is a lack of efficacy and safety data in a population of interest, pharmacokinetic data can be used to extrapolate efficacy from a population which has been studied, as has been proposed and is increasingly being used for pediatrics (6). Pharmacokinetic data can therefore be very useful to support medicine use in a new population. In light of the lack of efficacy data in pregnancy, we collected pharmacokinetic data for a number of medicines important for use during pregnancy, in order to consider whether this could be used to support posology, or possible dose adjustments, when use is clinically essential in this population.

The aim of this work was to determine the extent of literature information that was available for each medicine and to determine which medicines had rich data sets. This review evaluates the availability of published PK data in pregnancy for medicines commonly used by pregnant women and highlights some common issues that limit usefulness of existing data in this population.

## Methods

A number of therapeutic indications and particular medicines of interest for pregnancy were identified, with input from clinical assessors at the MHRA. Thereafter, further advice on the medicines list was sought from Independent Experts on the Commission on Human Medicines (CHM) and its Advisory committees, including the Medicines for Women's Health Expert Advisory Group (MWHEAG) and other relevant therapeutic area Expert Advisory Groups (EAGs).

The list (Table 1) comprised 214 drug, or drug combinations, and was used to drive literature searches which were aimed at collecting literature pharmacokinetic data in pregnancy for each medicine of interest. More detailed analysis of the PK data was conducted for a subset of the drugs and will be the focus of future publications.

**Table 1:** List of medicines commonly used in pregnancy in the UK.

**Anesthetics**	**Antidepressants (cont')**	***HIV (cont')***	**Diabetes medicines (cont')**
Etomidate	Sertraline	Darunavir/cobicistat	Tolbutamide
Ketamine	Tranylcypromine	Darunavir/ritonavir	**Endocrine medicines**
Propofol	Trazodone	Dolutegravir	Thyroxine
Thiopentone	Trimipramine	Doravirine	**Anti-epileptics**
**Antibiotics**	Triptafen	Efavirenz	Brivaracetam
Amikacin	Venlafaxine	Elvitegravir/cobicistat	Buprenorphine
Amoxicillin	Vortioxetine	Emtricitabine	Carbamazepine
Ampicillin	**Anti-emetics**	Lamivudine	Clobazam
Azithromycin	Cyclizine	Nevirapine	Clonazepam
Benzylpenicillin	Doxylamine/pyridoxine	Raltegravir	Diazepam
Cefaclor	Meclozine	Rilpivirine	Eslicarbazepine
Cefadroxil	Metoclopramide	Tenofovir alafenamide	Ethosuximide
Cefalexin	Ondansetron	Tenofovir DF	Felbamate
Cefixime	Phenothiazines	Zidovudine	Gabapentin
Cefotaxime	Promethazine	***HSV***	Lacosamide
Cefradrine	**Antifungal**	Acyclovir	Lamotrigine
Ceftazidime	Amphotericin	Valiciclovir	Levetiracetam
Ceftriaxone	Clotrimazole	***Influenza***	Lorazepam
Cefuroxime	**Antihistamines**	Oseltamivir	Midazolam
Clindamycin	Cetirizine	Zanamivir	Oxcarbazepine
Co-amoxiclav	Chlorpheniramine	**Cardiovascular medicines**	Perampanel
Co-fluampicil	Cyproheptadine	***Antihypertensives***	Phenobarbital
Ertapenem	Diphenhydramine	Atenolol	Phenytoin
Erythromycin	Hydroxyzine	Bisoprolol	Pregabalin
Flucloxacillin	Loratadine	Carvedilol	Primidone
Gentamicin	Promethazine	Digoxin	Rufinamide
Imipenem	Terfenadine	Flecainide	Stiripentol
Linezoil	Tripelennamine	Hydralazine (iv)	Tiagabine
Meropenem	**Anti-malarials**	Labetalol	Topiramate
Metronidazole	Artemether/lumefantrine	Methyldopa	Valproate
Nitrofurantoin	Artesunate	Metoprolol	Vigabatrin
Phenoxymethylpenicillin	Clindamycin	Nifedipine	Zonisamide
Piperacillin–tazobactam	Hydroxychloroquine	Propranolol	**Immunosuppressants**
Pivmecillinam	Quinine sulfate	Sildenafil	Azathioprine
Temocillin	**Antipsychotics**	Tadalafil	Cyclosporine
Trimethoprim	Clozapine	Verapamil	Interferon
Vancomycin/ teicoplanin	Risperidone	***Anti-thrombotics***	Prednisone
**Antidepressants**	**Anti-TB agents**	Aspirin	Sirolimus
Agomelatine	Bedaquiline	Dalteparin	Tacrolimus
Amitriptyline	Ethambutol	Enoxaparin	**Pain** medicines
Chlopromazine	Isoniazid	Nadroparin	Caffeine
Citalopram	Pyrazinamide	Tinzaparin	Codeine
Clomipramine	Rifampicin	VKA (warfarin)	Diamorphine
Dosulepin	**Antivirals**	**Chemotherapy**	Diclofenac
Doxepin	***HBV***	Dacabarzine	Dihydrocodeine
Duloxetine	Entecavir	**Corticosteroids**	Eletriptan
Escitalopram	TDF/TAF	Betamethasone	Entonox
Fluoxetine	***HCV***	Dexamethasone	Fentanyl
Fluvoxamine	Dasabuvir	Hydrocortisone	Gabapentin
Imipramine	Elbasvir	Methylprednisone	Ibuprofen
Isocarboxazid	Grazoprevir	Prednisone	Morphine
Lithium	Ledipasvir	**Diabetes medicines**	Paracetamol
Mianserin	Pibrentasvir	Chlorpropamide	Pethidine
Mirtazapine	Sofosbuvir	Glibenclamide	Rizatriptan
Moclobemide	Voxilaprevir	Glimepiride	Sumatriptan
Nortriptyline	***HIV***	Glipizide	Tramadol
Paroxetine	Abacavir	Glyburide	Zolmitriptan
Phenelzine	Atazanavir/cobicistat	Insulin	**Sedatives**
Quetiapine	Atazanavir/ritonavir	Metformin	Midazolam
Reboxetine	Bictegravir	Tolazamide	

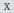
 Medicines group.

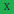
 Rich data.

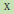
 Moderate/low data.

X No data.

The focus was on maternal exposure, and publicly available pregnancy data up to 2019 have been collected from the literature, clinical trial databases or published assessment reports for Regulatory submissions. PubMed and SciFinder searches were conducted using the search terms “pharmacokinetics of *medicine name* in pregnant women” within the abstract or title of the published papers. In addition, the clinical trial databases i.e., www.clinicaltrials.gov and were searched using the terms “*medicine name* AND pregnancy.” Papers were selected for review if they were published in English and focused on the evaluation and/or collection of PK data in at least one gestational trimester in women. Published studies with data on PD, safety and efficacy in pregnant population and data on fetal exposure or transfer into breastmilk were noted but not included here as these were out of scope for this review.

Information was captured on whether the study included pre- or postpartum data in the same individuals or if comparisons were made to data from non-pregnant individuals in other clinical studies. Likewise, information was collected on whether or not protein binding was considered, where this was known from non-pregnant individuals to influence PK significantly.

Generally, PK parameters are reported using standard compartmental analysis however in some cases, where data is sparse, population PK (PopPK) modeling was used. This modeling approach is, in general, used to characterize PK in a typical individual and to study the variability in drug concentrations between subjects within the population and considering individual characteristics such as age, sex, weight or disease state (7). For the purposes of this review, studies which collected data for PopPK modeling were included as available data. However, details on the PK models used to describe the data, e.g., PopPK and Physiologically Based Pharmacokinetic Modeling (PBPK) are not included here.

To obtain an overall picture of the availability of pregnancy PK data, each medicine was classified according to the quantity of available data, taking into account the abundance of information, the availability of data across the three trimesters and the richness and quality of the data. Table 2 outlines the aspects that were considered when evaluating the richness and quality of the data for each medicine.

**Table 2:** Study design methodology factors which may affect reliability of PK studies.

Exposure data at different stages of pregnancy	A change in blood levels related to pregnancy can be obscured by variability between subjects. Data from the same individual at different stages of pregnancy reduces overall variability as this allows each person to serve as their own control.
Controls vs non-pregnant data from the same individuals	Is non-pregnant data available in the same individuals' preconception or postpartum? Where data is compared with different non-pregnant subjects this will introduce inter-subject variability on the data. This introduces increased variability and reduces confidence in the conclusions. Comparing data from the same individual can indicate size and direction of pregnancy related changes even if there is larger variation between subjects. If postpartum values are used, what was the time point? It is important to consider whether sufficient time has elapsed for physiology to have returned to pre-pregnancy values in order to determine if the values represent truly non-pregnant values.
Number of subjects	Whether or not the number of subjects is sufficient to allow a good understanding of differences between pregnant and non-pregnant individuals will depend on the variability of the data. Variability in data captured was considered *via* means and standard deviations, CVs, or p-values (where tested). Larger variability between individuals requires larger numbers of subjects (unless subjects serve as their own controls), whereas smaller numbers of subjects may be sufficient to detect large pregnancy-related differences.
Doses and dose adjustments	How accurately are the doses used and any dose adjustments reported? Accurate reporting is important especially if different doses are used between individuals or at different stages of pregnancy. In some cases this may not be known or adjustments made but timing uncertain.
Dose route and formulation	Intravenous or oral dosing can give quite different profiles, in addition the oral formulation, whether it be immediate release or modified release, should be considered as this will influence the shape of the plasma concentration profile (e.g., venlafaxine) and will influence the PK parameters collected
Quantity and distribution of blood sampling times	Many sampling points allows a good understanding of the PK profile and changes in important parameters like Volume of distribution, Clearance and elimination half-life. Infrequent sampling around time of exposure may mean peak exposure levels are missed. Whereas studies with sparse opportunistic samples may only allow an understanding of the overall change in concentration, if used in a well characterized population PK model, this data can be used to inform changes in parameters. However, variability of concentrations may be exacerbated if timing of sampling in relation to dose is not accurately recorded.
Bioanalytical methodology	Good bioanalytical methodology is also important to support the reliability of the data. Ideally, publications should clarify the lowest level of drug that can be reliably detected. Conclusions on the data may be compromised if the method does not have an adequate detection sensitivity to allow a BLQ at least 10 fold below the levels being measured. The method also needs to be specific for the entity of interest, in some cases the drug may be a chiral mixture, so it is important to consider what has been measured and how it relates to the active enantiomer, e.g., fluoxetine. In other cases there may be active metabolites that are also of interest, e.g., metropolol.
Free drug levels	What was the measured entity appropriate, e.g., total or free drug and is this appropriate ? Decreased drug protein binding is expected in pregnancy due to the decreased levels of plasma proteins such as albumin and α1-acid glycoprotein. Therefore, measurement of free drug is important to account for differences in protein binding. In some cases free drug is measured directly, alternatively this can be calculated based on measurement of binding affinity in the study subjects or in matched subjects.
Effects of disease state	Is the disease state captured and considered, e.g., patients vs. healthy pregnant individuals vs. healthy non-pregnant individuals, the effect of disease can be greater than the effect of pregnancy, e.g., systemic infection can have a greater affect on PK of antibiotics than pregnancy. This should be considered when comparing pregnant and non-pregnant data from different clinical studies.
Effect of polymorphisms on PK	It may be important to consider the phenotype of the subjects in the study and if this has been documented, particularly of enzymes involved in the clearance of the drugs. Markedly different changes in exposure can occur in subjects that are poor or rapid metabolisers and the changes in pregnancy can be different in these subjects, e.g., metoprolol.

Medicines with high quality data in all gestational trimesters were generally considered to have rich data and classified as high category, while medicines with high quality data in two trimesters, or with sparse samples in all trimesters, were classified as medium category. Medicines for which data was limited, usually referring to only one trimester and/or with only sparse sampling from a single trimester, were classified as low category.

## Results

**Figure 1:**
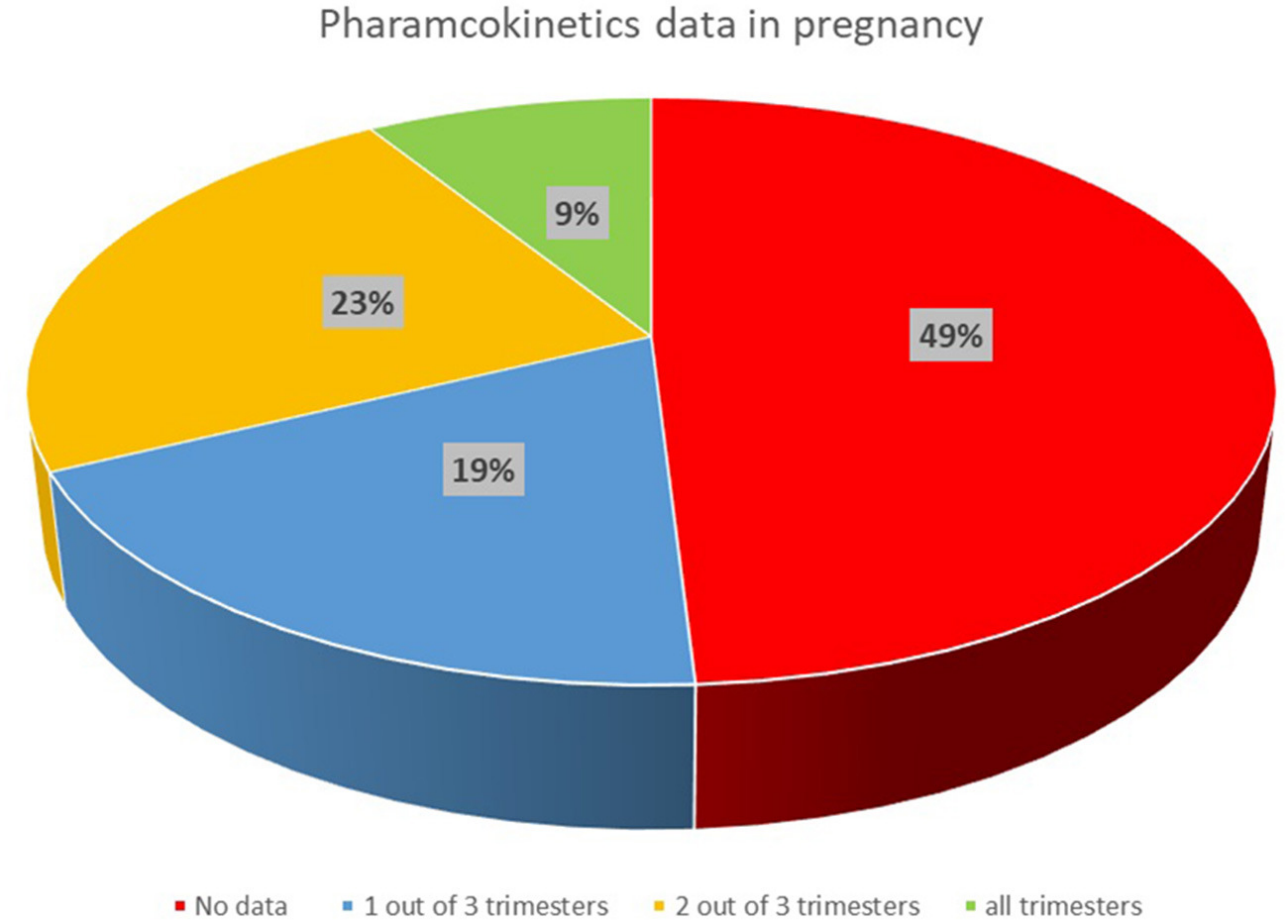
Availability of published clinical PK data in pregnancy for medicines of interest in the UK.

The literature searches were performed for the 214 drugs from the therapeutic areas identified to be of interest in pregnancy. Relevant PK data were identified for 109 out of 214 medicines, with no PK data identified for the remaining 105 drug searches. Therefore, available published PK data were found and collected for only approximately half of 214 medicines included in the search. Figure 1 shows the availability of published PK data, as percentage, for the medicines reported in Table 1.

Clinical PK data with relatively rich sampling were available for 19 out of 109 medicines in all gestational trimesters (high category), while data were available for 50 medicines in two trimesters or where only sparse samples were available in all of the trimesters, and only very limited data were collected for 40 out of 109 medicines.

In some cases, it was difficult to classify the availability of PK data when considering the balance of data quantity vs. quality. For example, the effect of pregnancy on the PK of clozapine and risperidone was investigated in one study only with sparse samples, so met the criteria for low PK data availability. However, the study appeared well designed and allowed the characterization of the expected PK changes in all gestational trimesters (8).

**Figure 2:**
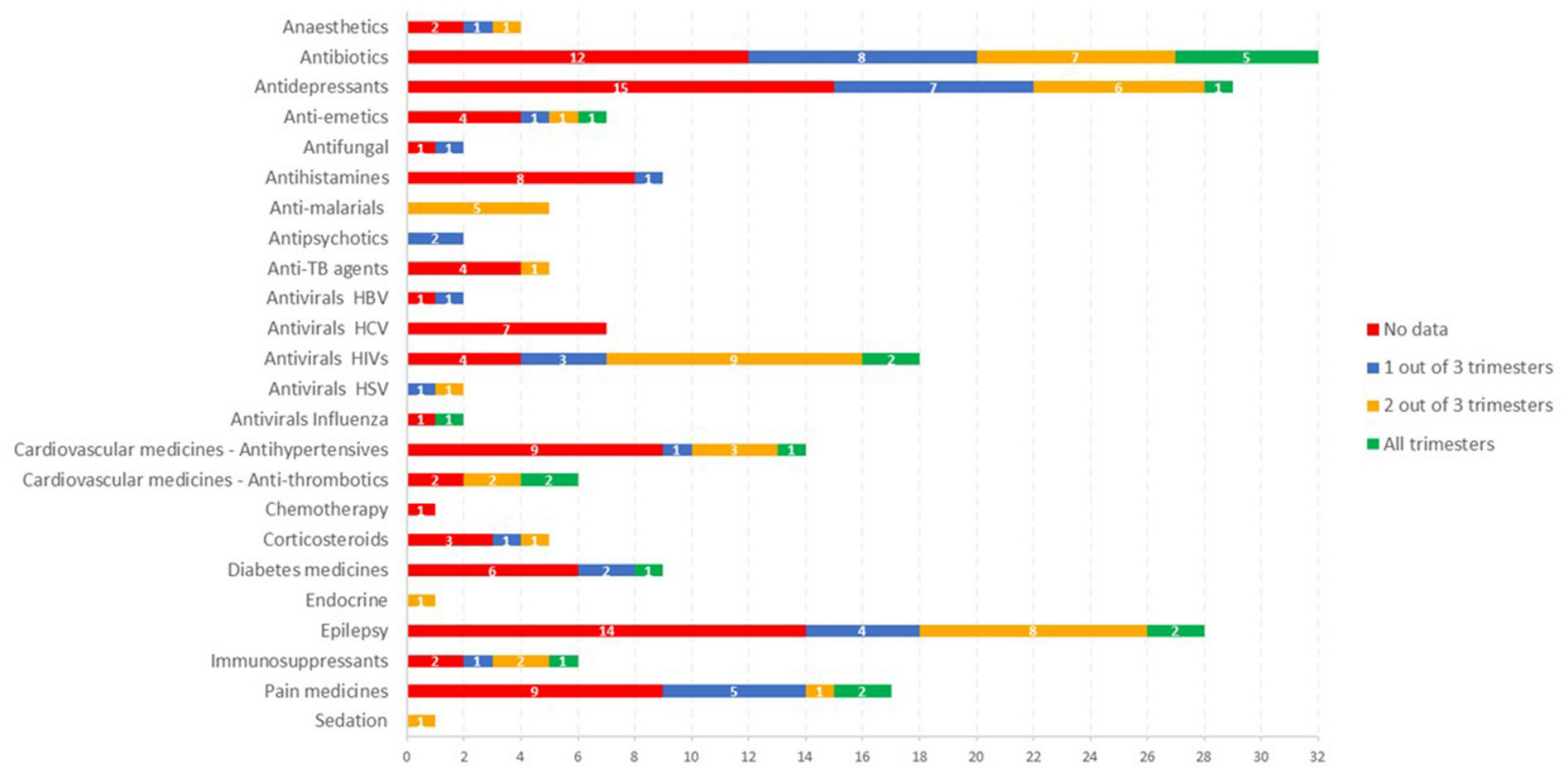
The availability of published PK data in pregnancy grouped by the investigated therapeutic areas.

In total over 420 publications with relevant data were identified. The amount of quality data available is very dependent on the therapeutic area, with some areas quite well studied in this population (e.g., antiviral (HIV), malaria, and antibiotics), whereas in other areas (e.g., antiemetics, TB, cardiovascular and diabetes), data is very limited, with most drugs having no data despite there being a need for treatment in this population. Figure 2 shows the availability of published PK data in pregnancy grouped by the investigated therapeutic areas.

In the antiviral area, HIV drugs are relatively well studied in pregnancy, with some data for most drugs and data is available across all trimesters for some medicines, e.g., atazanavir (9, 10). The availability of data in this area has been greatly facilitated by the IMPAACT study, which has studied at least 12 antiviral medicines in pregnancy using a platform study design.

For medicines in the antimalarial area, some data was available for all the drugs of interest (Figure 2) but in none of the cases were comprehensive data available across all trimesters. In some cases, the sparse data available was analyzed using a population PK analysis to show changes in pregnancy (12, 13).

For some antihypertensive medicines, some data was available for all trimesters, but full PK profiles were not available. Nevertheless, this allowed some conclusions on the impact of pregnancy compared with postpartum, e.g., with findings of up to 5 fold decreased exposure during pregnancy documented for metoprolol (14–16).

For medicines indicated for pain, there is currently reasonable data on the drugs blood levels in pregnancy for some drugs including, e.g., paracetamol (17–23) while limited PK data are available for other compounds in this therapeutic area (e.g., aspirin, morphine, tramadol, gabapentin, entonox, buprenorphine and opioids such as fentanyl and pethidine). However no PK data were identified in the pregnant population for some medicines, e.g., ibuprofen, triptans, codeine.

Corticosteroids as well as anti-hypertensives are commonly used during pregnancy. However, our search showed that the PK of those medicines' groups are not extensively studied in pregnancy i.e., PK data are not found for all pregnancy trimesters for medicines such as labetalol, nifedipine, dexamethasone or betamethasone. In other cases, e.g., methyldopa, PK data were not available in literature at the time of this review.

The PK of about half of the antidepressant drugs included in the search has been evaluated in pregnant women, although often a complete characterization of the PK profile is not available or data are not available for all gestational trimesters (e.g., citalopram, fluoxetine, sertraline, trazodone, lithium). Venlafaxine has been investigated in all trimesters of pregnancy, and decreased blood venlafaxine levels were observed in pregnancy compared to postpartum, although not all studies included postpartum measures or comparative data in non-pregnant individuals (24–26).

Amongst the immunosuppressant medicines, the PK of tacrolimus (11, 27, 28) has been investigated most extensively in pregnancy although the available data are not available in all gestational trimesters or consists of sparse samples. Of note, the available data suggest higher concentrations of free tacrolimus which may be clinically significant (11).

The impact of pregnancy on the PK of a number of antiepileptic drugs has been investigated by several authors. For some drugs (e.g., carbamazepine, phenytoin, levetiracetam, lamotrigine, valproate), PK data are available in all gestational trimesters, or at least two of them, although in general only sparse data have been collected (29–33). Significant decreases in exposure have been reported for lamotrigine (33, 34), whereas, although some authors reported a small reduction in total carbamazepine (11), the pharmacologically active free carbamazepine and its clearance did not, in general, significantly change during pregnancy (30–32).

Anesthetics (e.g., propofol, thiopentone) as well as sedative medicines (e.g., midazolam) do not appear to have been investigated during pregnancy, although some limited PK data are available in women undergoing cesarean section (35–38).

A limited number of studies were found which investigated the PK of antipsychotics (8, 9) in pregnancy, although in these studies data were collected in each gestational trimesters as well as at postpartum.

For diabetic medicines, the PK of metformin (39, 40) has been investigated during pregnancy, however limited data are available for other medicines commonly used, e.g., insulin and glyburide.

## Discussion

More than 80% of women take at least one medicine during pregnancy (41, 42) and recent estimates suggest that one in five UK pregnancies were reported to be affected by polypharmacy (taking two or more prescription medicines at the same time^1^. It is therefore important to understand the impact of pregnancy-related physiological changes on the systemic exposure of medicines. The collection of PK data to evaluate this can support the benefit-risk evaluations of medicines used in pregnancy and support requirements for dose selection. This review focusses on PK. Dose adjustments based solely on PK assume that there are no changes in PD in this population, which is not always the case, e.g., glycemia control, or blood pressure. This needs to be considered on an individual therapeutic basis.

Our review of the literature for pharmacokinetic data on a number of medicines of interest for use in pregnancy has highlighted significant shortfalls in the availability of data. However, where data is available, significant changes in exposure are seen with decreased exposure of up to 5 fold documented, e.g., metoprolol (14, 15) and lamotrigine (33, 34), while for other medicines no changes are seen, e.g., carbamazepine (30–32). Higher concentrations of free drug than total drug can be observed in pregnancy, and these changes may be clinically significant for medicines such as tacrolimus (11). For other medicines, a decrease in total drug levels may not be related to a decrease in unbound concentrations. For instance, some authors reported a small reduction in total carbamazepine (29), but the pharmacologically active free carbamazepine and its clearance did not, in general, significantly change during pregnancy (30–32). This highlights the importance of study design in evaluating PK. A well designed PK study with a rich sampling set allows a good understanding of the PK profile and changes in important parameters like Volume of distribution, Clearance and elimination half-life. Whereas studies with sparse opportunistic samples may only allow an understanding of the overall change in concentration. However even in the latter case, if used in a well characterized population PK model, this data can also be used to inform changes in parameters.

The differential changes in exposure levels underline why more studies are urgently needed to support dosing of medicinal products in this population. There is also generally a dearth of new medications to treat obstetrical and lactation disorders (1). Although much needed, treatments for obstetric complications appear underdeveloped compared with other fields of medicine, and the appetite of pharmaceutical companies to invest in research for obstetric syndromes is generally reduced by concerns for maternal, fetal, and infant safety, poor definition, and regulatory paths toward product approval (43). In addition to ethical considerations, extra logistical challenges exist for clinical studies in pregnant women that need to be addressed. Therefore, it is important to maximize the usefulness of studies in this population.

A number of limitations in existing study methodology were noted in the PK data collected and are summarized (Table 2), consideration of these can be used to influence the design and maximize the output from future studies in this population.

Variability in exposure levels and other PK parameters made it difficult to determine if and when changes related to pregnancy occur. In some studies, this was likely due to inclusion of too few participants, so the studies were underpowered to detect statistically significant changes. Studies should include a sufficient number of subjects to allow significant changes in exposure to be detected. Consideration of the variability of the drug in non-pregnant individuals can be used to inform the number of subjects, similar to the approach taken for pediatric studies (44, 45).

Information on time of sampling since dose was often poorly reported, especially for studies with sparse sampling. Inaccuracies in this can underestimate peak plasma levels and affect estimates of elimination time. Ideally, the drugs levels should be measured at several time points to allow an accurate characterization of the PK changes in pregnancy. If plasma samples cannot be collected at the exact scheduled time, it is important to record accurately information about the time of the sample collection in relation to when the drug was administered. Consideration should be given to the dose route and formulation when considering appropriateness of sample points, especially around time of expected peak plasma levels, since intravenous or oral dosing can give quite different profiles, and immediate or modified release formulations will influence the shape of the plasma concentration profile (e.g., venlafaxine).

A combination of sparse sampling and population PK modeling such as in the study by Karanam (46) can allow the characterization of PK changes in different gestational trimesters and allows the integration of other data to better inform changes in pregnancy. This suggests that more use could be made of opportunistic samples, particularly if it is difficult to obtain plasma samples so a rich PK dataset is not feasible. Since medical visits are usually frequent in pregnancy, opportunistic samples from routine blood collection for laboratory tests could be used for PK evaluation, provided good bioanalytical support is available. Such an approach should ideally collect at least some samples around expected Tmax and during the elimination phase to support the understanding of possible impact of pregnancy on the medicine absorption and elimination.

It is worth considering aspects specific to this population that are important when interpreting data from these studies. Changes in exposure with time as pregnancy progresses can be rapid whereas changes that occur with age are usually slow. Where large changes in exposure are expected or seen during pregnancy it is important to have data at early stages of pregnancy, e.g., first trimester and to have earlier time points post-partum, to determine the time away from, and to return, of normal physiology. Knowledge of expected changes due to physiological changes or PBPK models would support the design of studies. Few studies also considered the impact of disease and/or polymorphisms in drug metabolizing enzymes on the exposure, although these could differentially affect pregnancy-related changes in drug blood levels as was seen for metoprolol (16).

Several studies evaluated PK changes in pregnancy by the comparison of the drug systemic exposure to the systemic exposure at postpartum or pre-pregnancy. However, time since delivery varied widely in some studies from several days to months in different studies (or even within the same study) and was poorly reported in others. For true post-partum, for most medicines, data should be collected at least 6 weeks after delivery as the pregnancy-related physiological changes are expected to revert to the non-pregnant status (47, 48). Moreover, if possible, data should be collected in the same woman during both pregnancy and postpartum in order to reduce the possible effect of intersubject variability. Data from the same woman in multiple trimesters may only be feasible for chronic treatments. However, sampling during pregnancy and postpartum from the same individuals and collected from women at different trimesters should be feasible for acute or short term treatments. Population PK analysis could be used to characterize pregnancy related changes for medicines use in non-chronic conditions.

Alternative approaches include platform type trials, as used in Impaact which can be used to efficiently investigate a number of medicines in a particular therapeutic area, the use of innovative designs and complex trial designs has also been proposed by other publications (49), Alternatively it can be efficient to use existing data, e.g., from therapeutic monitoring programmes, to examine changes in exposure in this population.

The importance of a study with rich sampling over a study with sparse samples would depend on how well characterized the PK profile is in the non-pregnant population and the level of PD understanding. If a trough concentration is recognized as the important determination of efficacy, and there are limited safety concerns, then determination of the trough concentration could be sufficient, in other cases, where PKPD is less well understood, it would be important to characterize the whole profile (AUC, Cmax and T1/2) with a dedicated PK study with rich sampling or informed time points analyzed in conjunction with a PopPK model.

Generally, the integrated approach to support dosing in pregnancy, as has been advocated by other publications (50, 51), utilizing all available methodologies and supported by modeling is supported and would be considered to add great value to inform dosing in this population.

It is recognized that this study has some limitations. Papers without references to PK pregnancy data in either the title or abstract may not be included in the results of this search. Moreover, the search terms “blood/serum concentrations” were not used.

Cited references were selected as good example of the currently available information on the impact of pregnancy on the PK, or of the clinical study methodology. A full list of papers will be provided on the MHRA pregnancy website.

The data identified in this review spans some 35+ years of PK studies in pregnancy (30). Some of the limitations in reporting of key information may be related to age of the studies. Over this time period the CONSORT (http://www.consort-statement.org/) and STROBE (https://www.strobe-statement.org/) statements have improved reporting for clinical trials and observational studies respectively. There is currently no agreed standard for reporting of PK studies akin to these, likewise there is no regulatory guidance for the design of PK studies in pregnancy however guidance does exist for the reporting of PK studies in pediatrics (6, 45) and of population pharmacokinetics (52) for regulatory purposes. Whilst these provide useful guidance on considerations on the design of clinical studies, many studies are conducted by independent academics, so a statement on standards for publication of PK studies in scientific journals would help the design of efficient studies to provide valuable data.

In conclusion, pregnancy-related physiological changes are expected to alter the pharmacokinetics of medicines commonly used by pregnant women. Despite this, our review shows that very limited data are currently available to characterize the impact of those changes on the maternal blood levels of most medicines commonly used during pregnancy. An improved understanding of the pharmacokinetic changes in all gestational trimesters is urgently needed to aid the benefit-risk evaluations of medicines used by pregnant women and to support safe and effective doses in this population. When designing clinical studies there are a number of important considerations on the study methodology to maximize the value of the data and minimize the impact on subjects in the trials.

## Author contributions

All authors listed have made a substantial, direct, and intellectual contribution to the work and approved it for publication.

## Funding

The MHRA received a grant from the Bill & Melinda Gates Foundation, Seattle, Washington, United States, for a project to investigate the utilization of PK and PBPK to improve drug use in pregnancy.

## Conflict of interest

PC was an employee of the MHRA at the time of the research study and manuscript writing. She is currently an employee of AstraZeneca UK Ltd. The authors declare that this study received funding from the Bill and Melinda Gates Foundation. The funder was not involved in the study design, collection, analysis, interpretation of data, the writing of this article, and the decision to submit it for publication. The remaining authors declare that the research was conducted in the absence of any commercial or financial relationships that could be construed as a potential conflict of interest.

## Publisher's note

All claims expressed in this article are solely those of the authors and do not necessarily represent those of their affiliated organizations, or those of the publisher, the editors and the reviewers. Any product that may be evaluated in this article, or claim that may be made by its manufacturer, is not guaranteed or endorsed by the publisher.

## Author disclaimer

The views expressed in this article are the personal views of the authors and may not be understood or quoted as being made on behalf of or reflecting the position of the regulatory agencies or other organizations with which the authors are affiliated.

## Footnotes

1 https://mumpredict.org/what-is-polypharmacy/
